# Effect of interval between food intake and drug administration at fasting condition on the plasma concentrations of first-line anti-tuberculosis drugs in Chinese population

**DOI:** 10.1097/MD.0000000000022258

**Published:** 2020-10-30

**Authors:** Jun Wang, Jing Wang, Yadong Du, Ru Guo, Xiqin Han, Qingfeng Wang, Yu Pang, Naihui Chu

**Affiliations:** a Tuberculosis Department; b National Clinical Laboratory on Tuberculosis, Beijing Key Laboratory on Drug-Resistant Tuberculosis, Beijing Chest Hospital, Capital Medical University, Beijing Tuberculosis and Thoracic Tumor Research Institute, Beijing, China.

**Keywords:** drug, first-line, plasma concentrations, tuberculosis

## Abstract

We aimed to investigate the effect of interval between food intake and drug administration at fasting condition on the plasma concentrations of first-line anti- tuberculosis (TB) drugs in Chinese population. Newly diagnosed TB patients administered the anti-TB drugs under fasting conditions orally, and then had prepared breakfast at 30 minutes and 120 min after dosing, respectively. Blood sampling was also performed 120  minutes after dosing for the detection of *C*_max_ purpose. Overall, twenty-five participants were included in our analysis. The *C*_max_s of 30  minutes interval and 120  minutes interval were 21.8 ± 2.0 and 19.2 ± 2.0 μg/mL for rifampin, 1.6 ± 0.2 and 2.1 ± 0.2 μg/mL for isoniazid (INH), 1.5 ± 0.1and 1.5 ± 0.2 μg/mL for ethambutol (EMB), and 49.2 ± 3.7 and 41.5 ± 3.9 μg/mL for pyrazinamide, respectively. Statistical analysis revealed that there was no statistical difference between 2 groups. Additionally, 88.0% and 72.0% of the 25 participants at 2-hour interval group had peak concentrations less than the lower limit of the reference range for INH and EMB, respectively. The *C*_max_s of INH were 0.9 ± 0.4 μg/ml for rapid acetylator, which was significantly lower than those of intermediate (1.4 ± 1.0 μg/mL), and slow acetylator (2.5 ± 1.0 μg/mL), respectively (*P* < .01). In conclusion, our data demonstrate that early food intake at 30 minutes after drug administration had no significant influence on the plasma concentrations. In addition, a high proportion of patients receiving first-line anti-TB regimen fail to achieve the expected plasma drug ranges of INH and EMB (*P* > .05).

## 1 Introduction

Tuberculosis (TB), caused by Mycobacterium tuberculosis (MTB) complex, presents a great challenge to public health, especially for developing countries.^[1]^ In 2017, an estimated 10.0 million people developed TB and 1.6 million died because of TB globally.^[1]^ The control of TB depends on early diagnosis and effective treatment of the infectious patients.^[2]^ The majority of TB patients are affected by drug-susceptible tubercle bacilli, and strong evidence has demonstrated that standardized treatment regimens with first-line drugs, consisting of isoniazid (INH), rifampin (RIF), ethambutol (EMB) and pyrazinamide (PZA), are highly successful in drug-susceptible TB.^[3]^ Reported treatment success rates range from 60% to 90% depending on comorbidities and patient adherence.^[4]^ Efforts to improve treatment outcomes requires a better understanding of the factors contributing to the TB patients’ adherence.^[5]^

Adherence to the long course of anti-TB treatment is influenced by a wide range of factors.^[5]^ Patient non-adherence is majorly due to adverse drug events associated with treatment mediation.^[6]^ Many of anti-TB drugs can lead to gastrointestinal upset, such as nausea, vomiting and abdominal pain.^[7]^ Previous studies revealed that the interactions between food and drugs could reduce the bioavailability of anti-TB drugs, especially for RIF and INH.^[4]^ As a consequence, TB patients are endorsed to take anti-TB drugs at fasting condition to avoid therapeutic failure due to reduced blood concentrations.^[8]^ Considering that food intake is responsible for reducing mucosal injury in the stomach, the drug administration after overnight fasting would accelerate the occurrence of gastrointestinal upset among individuals with gastrointestinal intolerance.^[9]^ Thus, the question raised by this dilemma is to determine the optimal timing of taking anti-TB drugs to balance gastrointestinal upset and effective blood concentrations. Unfortunately, previous studies were often focused on the influence of concomitant intake food on anti-TB drug concentrations.^[4,7]^ Therefore, the goal of this study was to investigate the effect of interval between food intake and drug administration at fasting condition on the plasma concentrations of first-line anti-TB drugs in Chinese population.

## 2 Patients and methods

### 2.1 Study design and population

From January 2018 through February 2019, newly diagnosed TB patients in Beijing, China, who were > 18 years old were asked to participate in the study. This study was approved by the Ethics Committees of the Beijing Chest Hospital, Capital Medical University. All the patients enrolled in the study provided written informed consent.

Study subjects had positive smear microcopy and/or GeneXpert results, and were administered the conventional daily 2-month intensive phase of the 4-drug anti-TB regimen, consisting of 300 mg INH (Hongqi Pharmaceutical Co., Shenyang, China), 600 mg RIF (Tongde Pharmaceutical Co., Chengdu, China), 750 mg EMB (Jinhua Pharmaceutical Co., Chengdu, China), and 1500 mg PZA (Jinhua Pharmaceutical Co., Chengdu, China).

Patients with any of the following conditions were excluded from the present study:

(1) human immunodeficiency virus infection;
(2) pregnancy;
(3) known drug resistance;
(4) diabetes mellitus;
(5) renal or hepatic disease;
(6) intolerance to any first-line drug;
(7) chronic alcohol consumption;
(8) gastrointestinal disease.

The demographic data of eligible patients were obtained from electronic medical records. Characteristics studied included age, sex, weight, baseline liver function, and blood glucose.

### 2.2 Pharmacokinetics

The anti-TB drugs were administrated under fasting conditions. It is routinely advised that patients must be fasted within 120 minutes after anti-TB administration, setting as control group, while the interval of 30 minutes was used as experimental group in this study. Prior to our analysis, all patients experienced a 6-day course of anti-TB drug administration. On days 7 and 8, they administered the anti-TB drugs orally, and then had prepared breakfast at 30 min and 120 min after dosing, respectively. Blood sampling was also performed 120 min after dosing for the detection of *C*_max_ purpose. Reference ranges for INH, RIF, EMB, and PZA were 3 to 6, 8 to 24, 2 to 6, and 20 to 60 μg/mL, respectively. The blood samples were centrifugated at 2000 rpm for 10 minutes, and plasma supernatant was removed and stored at -70°C refrigerator untill bioanalysis. The plasma samples were analysed by liquid chromatography-tandem mass spectrometry method. Briefly, the separation of anti-TB drugs was conducted using Zorbax SB-Aq column (Agilent, 50mm × 4.6 mm, 5 μm) (Agilent Technologies, Waldbronn, Germany). The mobile phase was composed of 5 mM ammonium acetate (phase A) and 90% (v/v) acetonitrile in water containing 0.1% (v/v) formic acid (phase B) delivered in a gradient elution mode at 0.3 mL/min: phase B increased linearly from 1.0 to 100.0% from 0 to 12 min. The concentrations of anti-TB drugs were detected by Agilent 6400 triple quadrupole mass spectrometer (Agilent Technologies, Waldbronn, Germany). The method has been validated following US FDA guidance for industry on bioanalytical method validation.

### 2.3 Genotyping

Genomic DeoxyriboNucleic Acid (DNA) from the peripheral blood samples was extracted using the Lab-Aid Kit (Zeesan Biotech Co., Xiamen, China) according to the manufacturer's instruction.^[10]^ The isolated genomic DNA samples were genotyped by the multicolor melting curve analysis genotyping method (Zeesan Biotech Co., Xiamen, China). Briefly, the reaction mixture was prepared in a total volume of 25 μL, consisting of 19.6 μL of polymerase chain reaction (PCR) master mix, 0.4 μL Taq polymerase and 5 μL of template DNA. The PCR was conducted under the following conditions: initial denaturation at 95°C for 10 minutes, 10 cycles of denaturation at 95°C for 15 seconds, 70°C for 15 seconds with 1°C decrease per cycle until the temperature reached 60°C, extension at 76°C for 20 s, and then 50 cycles of denaturation at 95°C for 15 seconds, annealing at 60°C for 15 seconds and extension at 76°C for 20 seconds. After PCR amplification, the multicolor melting curve analysis genotyping was carried out under the following conditions: denaturation at 95°C for 1 minutes, renaturation at 35°C for 3  minutes, and a continuous record of fluorescence from 45 to 85°C. The patients were classified as rapid, intermediate, and slow acetylator phenotype according to the established criteria for the 4-single nucleotide polymorphism genotype panel.^[11]^

### 2.4 Statistical analysis

For each patient, the *C*_max_ was defined as the concentration measured at 120 minutes.^[12]^ The Student *t* test or Wilcoxon rank-sum test was used for the comparison of plasma drug concentrations. Two-sided *P* values less than .05 were considered significant. All statistical analysis was performed with SPSS Statistics 20.0 (SPSS Inc, Chicago, IL).

## 3 Results

Between January 2018 and February 2019, a total of 60 patients were included at enrollment. Among these patients, 20 cases were excluded due to adverse events associated with anti-TB drug administration; 13 were infected with RIF-resistant TB; and 2 withdrawed during study period at their own request. Finally, 25 participants were included in the study. The characteristics of patients were summarized in Table 1. The mean age of participants was 33.4 ± 7.1 years; 48% were male and 52% were female. The baseline levels of serum transaminases, creatinine, and blood glucose of all participants were within the normal ranges, respectively. By using the 4- single nucleotide polymorphism genotype panel for N-acetyltransferase 2 gene, 7 (28.0%) patients were rapid acetylators, 7 (28.0%) were intermediate acetylators, and 11 (44.0%) were slow acetylators for INH, respectively.

**Table 1:**
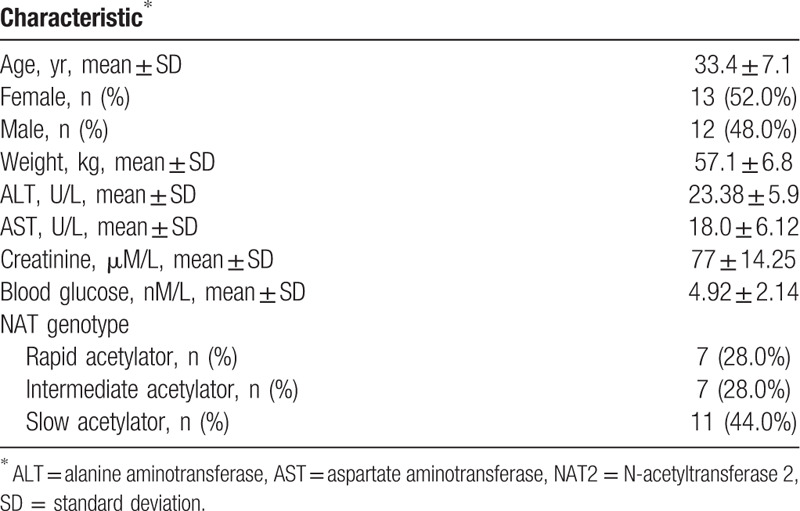
Demographic and clinical characteristics of patients enrolled in this study.

**Figure 1:**
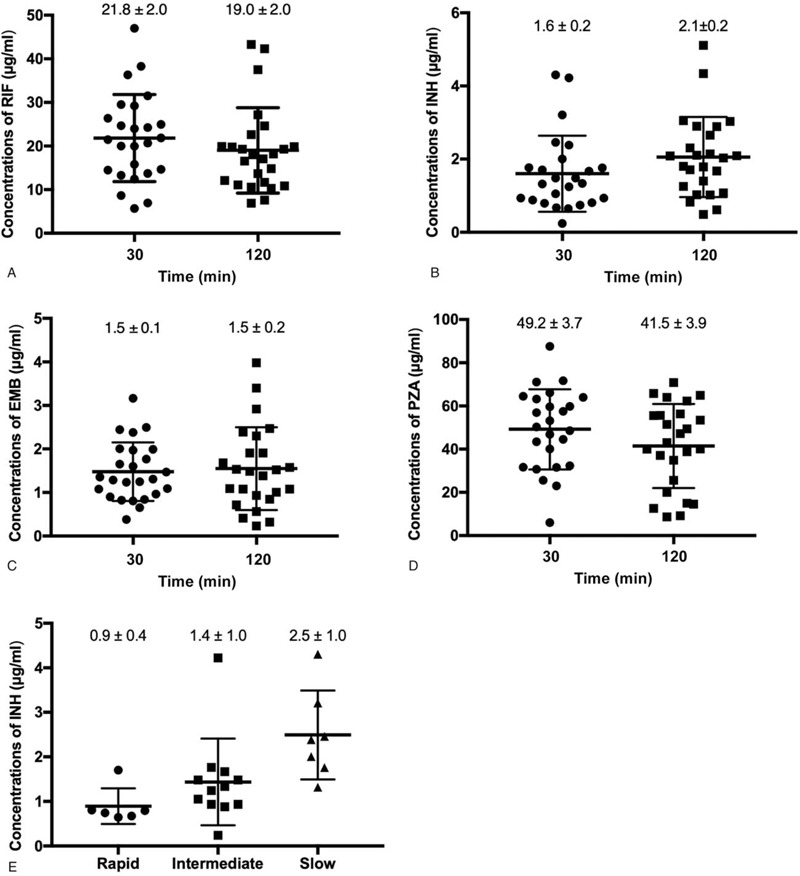
*C*_max_s of the first-line anti-TB drugs of different intervals between food intake and drug administration at fasting condition.

**Figure 2:**
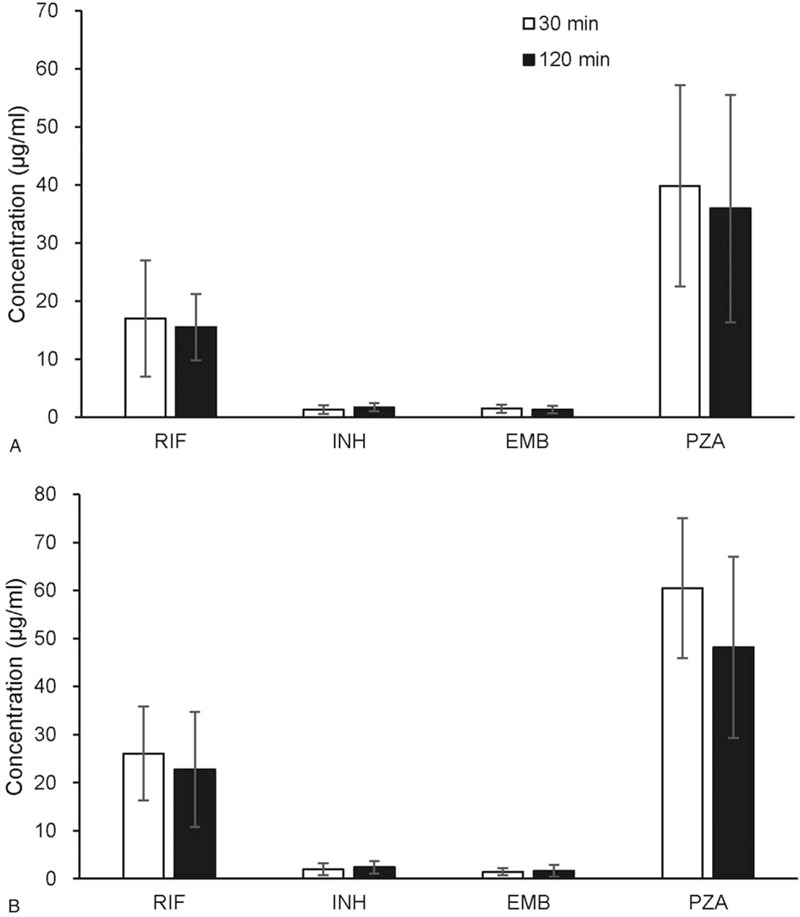
Comparison of *C*_max_s of the first-line anti-TB drugs stratified to sex. A, Comparison of *C*_max_s of the first-line anti-TB drugs between 30 minute interval and 120 minute interval in male patients. B, Comparison of *C*_max_s of the first-line anti-TB drugs between 30 minute interval and 120 minute interval in female patients.

In Figure 1, the *C*_max_s of the first-line anti-TB drugs of different intervals between food intake and drug administration at fasting condition are presented. The *C*_max_s of 30 minutes interval and 120 minutes interval were 21.8 ± 2.0 and 19.2 ± 2.0 μg/mL for RIF, 1.6 ± 0.2 and 2.1 ± 0.2 μg/mL for INH, 1.5 ± 0.1and 1.5 ± 0.2 μg/mL for EMB, and 49.2 ± 3.7 and 41.5 ± 3.9 μg/mL for PZA, respectively. Statistical analysis revealed that there was no statistical difference between 2 groups (*P* > .05). Notably, we observed that 22 (88.0%) and 18 (72.0%) of the 25 participants at 120 minutes interval group had peak concentrations less than the lower limit of the reference range for INH and EMB, respectively. In addition, sex had no significant difference on the *C*_max_s between 30 minutes interval and 120 minutes interval group (Fig. 2).

In addition, we analysed the *C*_max_ of patients stratified to acetylator phenotype for INH in the 120  minute interval group. As shown in Figure 1E, the *C*_max_s of INH were 0.9 ± 0.4 μg/ml for rapid acetylator, which was significantly lower than those of intermediate (1.4 ± 1.0 μg/mL), and slow acetylator (2.5 ± 1.0 μg/mL), respectively (*P* < .01).

## 4 Discussion

In view of the fact that fed state decreases the absolute bioavailability of INH and RIF,^[7]^ the current guidelines on TB treatment recommend the administration of first-line anti-TB drugs at fasting condition. For patients undergoing gastrointestinal intolerance, the timing of the drug administration should be changed, preferably to be closer to mealtime. However, no evidence is provided. In Taiwan, random medication was even proposed to improve patient compliance. Thus, to achieve adequate serum drug concentrations, clinicians should be aware of the timing of anti-TB drug administration in these patients. In this study, we have described the plasma concentrations of first-line anti-TB drugs among the patients with different intervals between food intake and drug administration at fasting condition in Chinese population. Our data showed that early food intake after drug administration had no significant influence on the plasma concentrations of first-line anti-TB drugs (30 min versus 120 min). Previous experimental studies indicated that the components of the diet can majorly influence the development of injury in different regions of the gastrointestinal mucosa.^[9]^ The earlier food intake after administration will contribute to the decreased incidence of gastrointestinal adverse events. Another concern raised is whether the food intake would affect the drug dissolution, drug stability and intestinal permeability.^[9]^ Our results suggest that half an hour is potentially adequate for the completion of drug absorption for first-line anti-TB drugs. Notably, in many programs conducted in the TB high-burden settings, food is offered as an incentive with directly observed treatment, which is co-administrated with anti-TB drugs.^[8]^ In view of the reduced peak concentrations of drugs by food, the incentive food should be given at least 30 min after drug intake.

Another noteworthy finding of this study is that a high proportion of patients receiving first-line anti-TB regimen failed to achieve the expected plasma drug ranges of INH and EMB, respectively. In line with our results, Tappero et al. found that low concentrations were more frequently observed in INH and EMB.^[12]^ Furthermore, a previous study by Choudri and colleagues revealed that 89% of patients tested had peak INH levels less than 3 μg/ml, similar to 88% observed in our Chinese population,^[13]^ whereas the conflicting results were reported by McIlleron that indicated only 2% of participants had peak concentrations less than expected range.^[14]^ We speculate the frequencies of N-acetyltransferase 2 polymorphisms that vary widely between different population may be the major reason for the corresponding differences in INH pharmacokinetics across various studies.^[15]^ There is strong evidence demonstrating that the low 120 min INH concentration is associated with the poor treatment outcomes among TB patients.^[16]^ Hence, the INH plasma concentration of TB patient should be monitored at during the initial therapy period to formulate the optimal dosage of INH in the regimen. In addition, the low EMB level was noted in 72% of TB patients in this study. Although EMB is not as potent as INH and RIF, the lowered maximum concentration may increase the risk of acquired drug resistance. The high incidence of low EMB concentrations in Chinese population highlights that the EMB dosage should be properly increased to achieve the recommended concentrations.

We acknowledge that this study had several obvious limitations. First, the prepared breakfast was taken by participants, and we could not assess whether food component has a significant effect on the bioavailability of anti-TB drugs in the present study. Second, only 1 120 minute blood sample was collected from each participant. It is unable to calculate the other pharmacokinetics parameters. Third, a high rate of patients experiencing early treatment termination due to adverse events were excluded from our analysis. The high plasma concentration may be an important reason for emergence of adverse events. Hence, the exclusion of these patients may lead to underestimation of plasma concentrations in TB patients. Finally, the naïve patients rather than those with comorbidities were enrolled in this study. Previous studies have indicated that the comorbidities may affect the drug absorption and bio-distribution.^[17]^ Further studies are needed to evaluate the effect of intervals between food intake and drug administration on plasma drug concentrations in these special populations.

To conclude, our data demonstrate that early food intake at 30 minute after drug administration had no significant influence on the plasma concentrations of first-line anti-TB drugs. In addition, a high proportion of patients receiving first-line anti-TB regimen fail to achieve the expected plasma drug ranges of INH and EMB. The high incidence of low drug concentrations in Chinese population highlights that the INH and EMB dosage should be properly increased to achieve the recommended concentrations. Further studies are needed to confirm these results in clinical trials for patients taking first-line drugs with meals at different intervals.

## Author contributions

**Conceptualization:** Naihui Chu, Yu Pang.

**Data curation:** Jun Wang, Jing Wang, Yadong Du, Ru Guo, Xiqin Han, Qingfeng Wang.

**Formal analysis:** Jun Wang, Jing Wang, Yadong Du, Ru Guo, Xiqin Han.

**Investigation:** Jun Wang, Jing Wang, Yadong Du, Ru Guo, Qingfeng Wang.

**Supervision:** Qingfeng Wang, Naihui Chu, Yu Pang.

**Writing – original draft:** Jun Wang, Jing Wang.

**Writing – review & editing:** Naihui Chu, Yu Pang.

## References

[1] Word Health Organization. Global tuberculosis report 2018. Geneva: Word Health Organization; 2018.

[2] Zumla A Chakaya J Centis R Tuberculosis treatment and management--an update on treatment regimens, trials, new drugs, and adjunct therapies. Lancet Respir Med 2015;3:220–34. 25773212 10.1016/S2213-2600(15)00063-6

[3] Ma Z Lienhardt C McIlleron H Global tuberculosis drug development pipeline: the need and the reality. Lancet 2010;375:2100–9. 20488518 10.1016/S0140-6736(10)60359-9

[4] Saktiawati AM Sturkenboom MG Stienstra Y Impact of food on the pharmacokinetics of first-line anti-TB drugs in treatment-naive TB patients: a randomized cross-over trial. J Antimicrob Chemother 2016;71:703–10. 26661397 10.1093/jac/dkv394

[5] Munro SA Lewin SA Smith HJ Patient adherence to tuberculosis treatment: a systematic review of qualitative research. PLoS Med 2007;4:e238. 17676945 10.1371/journal.pmed.0040238 PMC1925126

[6] Watkins RE Rouse CR Plant AJ Tuberculosis treatment delivery in Bali: a qualitative study of clinic staff perceptions. Int J Tuberc Lung Dis 2004;8:218–25. 15139451

[7] Lin MY Lin SJ Chan LC Impact of food and antacids on the pharmacokinetics of anti-tuberculosis drugs: systematic review and meta-analysis. Int J Tuberc Lung Dis 2010;14:806–18. 20550762

[8] Blumberg HM Burman WJ Chaisson RE American Thoracic Society/Centers for Disease Control and Prevention/Infectious Diseases Society of America: treatment of tuberculosis. Am J Respir Crit Care Med 2003;167:603–62. 12588714 10.1164/rccm.167.4.603

[9] Rainsford KD Bjarnason I NSAIDs: take with food or after fasting? J Pharm Pharmacol 2012;64: 465–9. 22420652 10.1111/j.2042-7158.2011.01406.x

[10] Hu Y Chen S Yu X Rapid identification of the NAT2 genotype in tuberculosis patients by multicolor melting curve analysis Pharmacogenomics. Pharmacogenomics 2016;17:1211–8. 27377479 10.2217/pgs-2016-0026

[11] Dompreh A Tang X Zhou J Effect of genetic variation of NAT2 on isoniazid and SLCO1B1 and CES2 on rifampin pharmacokinetics in ghanaian children with tuberculosis. Antimicrob Agents Chemother 2018 62. 10.1128/AAC.02099-17 PMC5826147 29263072

[12] Tappero JW Bradford WZ Agerton TB Serum concentrations of antimycobacterial drugs in patients with pulmonary tuberculosis in Botswana. Clin Infect Dis 2005;41:461–9. 16028152 10.1086/431984

[13] Choudhri SH Hawken M Gathua S Pharmacokinetics of antimycobacterial drugs in patients with tuberculosis. Clin Infect Dis 1997;25:104–11. 9243044 10.1086/514513

[14] McIlleron H Wash P Burger A Determinants of rifampin, isoniazid, pyrazinamide, and ethambutol pharmacokinetics in a cohort of tuberculosis patients. Antimicrob Agents Chemother 2006;50:1170–7. 16569826 10.1128/AAC.50.4.1170-1177.2006 PMC1426981

[15] McIlleron H Willemse M Werely CJ Isoniazid plasma concentrations in a cohort of South African children with tuberculosis: implications for international pediatric dosing guidelines. Clin Infect Dis 2009;48:1547–53. 19392636 10.1086/598192

[16] Weiner M Burman W Vernon A Low isoniazid concentrations and outcome of tuberculosis treatment with once-weekly isoniazid and rifapentine. Am J Respir Crit Care Med 2003;167:1341–7. 12531776 10.1164/rccm.200208-951OC

[17] Polasa K Murthy KJ Krishnaswamy K Rifampicin kinetics in undernutrition. Br J Clin Pharmacol 1984;17:481–4. 6721995 10.1111/j.1365-2125.1984.tb02377.x PMC1463407

